# Cell Death by SecTRAPs: Thioredoxin Reductase as a Prooxidant Killer of Cells

**DOI:** 10.1371/journal.pone.0001846

**Published:** 2008-04-02

**Authors:** Karin Anestål, Stefanie Prast-Nielsen, Narimantas Cenas, Elias S. J. Arnér

**Affiliations:** 1 Medical Nobel Institute for Biochemistry, Department of Medical Biochemistry and Biophysics, Karolinska Institutet, Stockholm, Sweden; 2 Institute of Biochemistry, Vilnius, Lithuania; University of Hong Kong, China

## Abstract

**Background:**

SecTRAPs (selenium compromised thioredoxin reductase-derived apoptotic proteins) can be formed from the selenoprotein thioredoxin reductase (TrxR) by targeting of its selenocysteine (Sec) residue with electrophiles, or by its removal through C-terminal truncation. SecTRAPs are devoid of thioredoxin reductase activity but can induce rapid cell death in cultured cancer cell lines by a gain of function.

**Principal Findings:**

Both human and rat SecTRAPs killed human A549 and HeLa cells. The cell death displayed both apoptotic and necrotic features. It did not require novel protein synthesis nor did it show extensive nuclear fragmentation, but it was attenuated by use of caspase inhibitors. The redox active disulfide/dithiol motif in the N-terminal domain of TrxR had to be maintained for manifestation of SecTRAP cytotoxicity. Stopped-flow kinetics showed that NADPH can reduce the FAD moiety in SecTRAPs at similar rates as in native TrxR and purified SecTRAPs could maintain NADPH oxidase activity, which was accelerated by low molecular weight substrates such as juglone. In a cellular context, SecTRAPs triggered extensive formation of reactive oxygen species (ROS) and consequently antioxidants could protect against the cell killing by SecTRAPs.

**Conclusions:**

We conclude that formation of SecTRAPs could contribute to the cytotoxicity seen upon exposure of cells to electrophilic agents targeting TrxR. SecTRAPs are prooxidant killers of cells, triggering mechanisms beyond those of a mere loss of thioredoxin reductase activity.

## Introduction

Mammalian thioredoxin reductases (TrxR, E.C. 1.8.1.9.) are selenoproteins, i.e. they belong to the unique family of proteins that contain a selenocysteine (Sec, U in one-letter code) residue [1]–[3]. TrxR has, together with the principal substrate thioredoxin (Trx), a wide range of functions in cells as a major reducing system for DNA synthesis, redox regulatory functions and antioxidant defense [4]–[9]. Three mammalian isoenzymes of TrxR have been identified, namely the most abundant predominantly cytosolic TrxR1 [1], [3], mitochondrial TrxR2 [10]–[12] and TGR (thioredoxin glutathione reductase), the latter mainly expressed in testis [13], [14]. It should be noted, in the context of this study, that TrxR proteins of other organisms such as bacteria, archaea, plants or insects, are typically not selenoproteins. There is also a lack of consensus for nomenclature of TrxR, sometimes abbreviated as TR or TXNRD, with additional abbreviations occurring, e.g. mitochondrial TrxR2 is the same enzyme as TR3 and TGR has also been called TR2.

Mammalian TrxR1 is a homodimeric protein with the two subunits arranged head to tail [15]. The first phases of the catalytic cycle involve a transfer of electrons from NADPH via an enzyme-bound FAD to a disulfide in the CVNVGC motif located in the N-terminal domain, which is highly homologous to the mechanisms of glutathione reductase and other members of the enzyme family [15]–[19]. The electrons in TrxR1 are subsequently transferred from the dithiol of the reduced CVNVGC motif to a selenenylsulfide within the C-terminal -GCUG motif of the other subunit in the dimeric holoenzyme. The selenenylsulfide is thereby reduced to a selenolthiol, which can finally reduce the disulfide in the active site of Trx or other substrates of TrxR [15], [17], [18], [20]. Alternative substrates for TrxR in addition to Trx encompass additional protein disulfide substrates [21], [22] as well as several low molecular weight compounds, such as selenite [23], lipoic acid [24], ascorbate [25] or quinones [26], [27].

Sec is a selenium-containing analog to cysteine but a stronger nucleophile with a low p*K*_a_, which makes Sec a highly reactive amino acid [28]. The reactivity of Sec is essential for the native catalytic activity of mammalian TrxR [18], [20]. However, the presence of Sec in TrxR at an easily accessible C-terminal position renders the enzyme highly susceptible to irreversible inhibition by derivatization of the Sec residue. Thus, TrxR can be inhibited by a wide range of electrophilic compounds, many of them used in anti-inflammatory or anticancer treatment. Such compounds include auranofin [29], arsenic trioxide [30], several alkylating quinones [27], platinum-containing anticancer drugs [31]–[34], and additional classical alkylating anticancer agents including nitrosourea [34], [35], melphalan and chlorambucil [34]. Endogenously produced electrophilic prostaglandin derivatives can also target the enzyme [36].

It is easily conceivable that an inhibitor of TrxR would impose a significant stress on cells due to the resulting inhibition of thioredoxin-dependent reactions, the outcome of which would depend upon the state and growth condition of the target cell [5], [6], [9]. In addition, we previously found that derivatization of the Sec residue in TrxR1 with electrophilic compounds may infer a gain of function to the protein as a potent and direct inducer of cell death [37]. A truncated form of TrxR1 lacking the two last amino acids (–Sec-Gly) also induced cell death when introduced into cells, while the full-length enzyme with normal enzymatic activity did not [37]. Cytotoxic effects previously described for TrxR1 in the form of GRIM-12 [38]–[42] seem to have been due to combinations of TrxR1-dependent p53 activation and apoptosis induced by Sec-deficient forms of TrxR1, as earlier discussed in detail [37].

The mechanism of the cell death triggered by cytotoxic forms of selenium compromised TrxR1-derived proteins has hitherto been unknown. In order to distinguish the unique properties of these proteins from those of native TrxR1 enzyme, we have named them SecTRAPs for selenium compromised thioredoxin reductase-derived apoptotic proteins, thereby referring to derivatives of mammalian TrxR that have *i,* a compromised Sec residue, *ii,* no Trx reducing capability and *iii,* the capacity to induce cell death by a gain of function. The term SecTRAP also refers to the “trap” for electrophilic compounds that the Sec residue in TrxR constitutes due to its high nucleophilicity. In the present study, we have analyzed the means by which SecTRAPs induce cell death.

We show here that not all forms of TrxR1 with a compromised Sec residue show cytotoxic properties as SecTRAPs. Only forms of the modified protein having an intact N-terminal CVNVGC motif and being able to propagate NADPH oxidase activity could induce cell death. This cell death had both apoptotic and necrotic features and it correlated to an increased intracellular oxidative stress. The findings suggest that the antioxidant selenoprotein TrxR1 under some circumstances can be converted into a potent prooxidant killer of cells.

## Materials and Methods

### Chemicals and reagents

The *BioPORTER* Quick easy protein delivery reagent was obtained from Gene therapy systems. Fetal calf serum (FCS) came from Biotech Line AS, whereas Dulbeccos modified Eagle medium, L-glutamine, phosphate buffered saline (PBS) and PCR-primers were from Invitrogen. Antibiotics were purchased from BIO-Whittaker Belgium. Ascorbic acid (Vit C), bovine serum albumin (BSA), Cycloheximide, 2′-(4-Ethoxyphenyl)-5-(4-methyl-1-piperazinyl)-2,5′-bi-1H-benzimidazole (Hoechst 33342), juglone, propidium iodide (PI), tumor necrosis factor-α (TNF-α), α-tocopherol (Vit E), 2′,7′-dichlorofluorescein (DCFH) and staurosporine (STS) came from Sigma-Aldrich Chemical Co. Cisplatin (Platinol®, cis-diamminedichloroplatinum; CDDP) came from Bristol Myers Squibb. zVAD-fmk was obtained from Promega, zDEVD-fmk from Biosource and zVDVAD-fmk from Calbiochem. AnnexinV-FITC fluorescence microscopy kit was purchased from BD Biosciences.

### Preparation of different forms of TrxR1 and SecTRAPs

The TrxR activity and estimated Sec content of the different TrxR1 and SecTRAP preparations used in this study are summarized in Table 1. The proteins were produced, purified and analyzed as follows.

**Table 1:** Properties of the protein preparations used in this study

Protein preparation	Specific activity a)	Estimated Sec content b)
	*units/mg*	*% of protein species*
Enzymatically active TrxR1 preparations		
Recombinant rat TrxR1 as produced	15–20	30–50
Recombinant full-length rat TrxR1	34	90–100
SecTRAP preparations		
rTrxR1-CDDP c,	<0.6	30–50
Truncated human TrxR1	<0.6	0
Truncated rat TrxR1	<0.6	0
Mutant TrxR1 preparations		
C59S/C64S mutant rat TrxR1	<0.6	30–50
C59S/C64S-CDDP d,	<0.6	30–50

a, The specific activity was determined with the standard DTNB assay [50] using 10 nM enzyme preparation.

b, Sec content was estimated from a combined assessment of specific activity, ^75^Se incorporation, production and purification method and comparisons to earlier determinations [43]–[46], [49].

c, Recombinant rat TrxR1 derivatized with cisplatin.

d, Mutant C59S/C64S rat TrxR1 derivatized with cisplatin.

#### Recombinant rat TrxR1

Recombinant rat TrxR1 was purified over 2′,5′-ADP-Sepharose (obtained from GE) from an overproducing *E.coli* system using BL21(DE3) cells co-transformed with the pET-TRS_TER_ and pSUABC plasmids, essentially as described previously [43], [44].

#### Full-length rat TrxR1

The full-length Sec-containing rat TrxR1 was enriched from the purified recombinant TrxR1 preparation using a phenyl arsine oxide (PAO) sepharose column as described elsewhere [45], [46].

#### Truncated rat TrxR1

The recombinant two-amino acid truncated rat TrxR1 (having the C-terminal motif –Gly-Cys-COOH instead of –Gly-Cys-Sec-Gly-COOH) was purified over 2′,5′-ADP-sepharose from an overproducing *E.coli* system using BL21(DE3) cells transformed with the pET-TR plasmid (without an engineered selenocysteine insertion sequence element), resulting in production of truncated enzyme as described previously [43]. For the determination of kinetic parameters, the enzyme was produced in a BL21(DE3) *gor* ^−^ strain kindly provided by Arne Holmgren, Karolinska Institutet.

#### Truncated human TrxR1

First, recombinant human TrxR1 was purified over 2′,5′-ADP-sepharose from an overproducing *E.coli* system using BL21(DE3) cells cotransformed with pSUABC and a pET24d(+) plasmid encoding the human TrxR1 open reading frame (isoform TXNRD1_v1 [47]) in fusion with a bacterial-type SECIS element (construct kindly provided from Dr. Antonio Miranda-Vizuete), essentially following the procedure described for expression and purification of recombinant rat TrxR1 (see above). The two-amino acid truncated human TrxR1 was subsequently collected from the initial flow-through fractions of a PAO-sepharose purification scheme [45], [46], using the purified recombinant human TrxR1 as starting material.

#### Rat C59S/C64S mutant TrxR1

The pET-TRS_TER_ plasmid [43] was used as template in a PCR reaction using the QuickChange site-Directed Mutagenesis Kit from Stratagen, using upper primer 1: TTTAGGTATG GAGCCCACGT TCACAGACGT TCCCCCG and complementary primer 2: CGGGGGAACGT CTGTGAACGT GGGCTCCATA CCTAAA. The PCR reaction was performed according to the protocol from Stratagene and the PCR product was transformed into DHL-α competent cells. A plasmid preparation containing the sequence-confirmed C59S/C64S construct was further used for a TSS transformation [48] together with pSUABC [43] into ORaa(DE3) cells [49]. Colonies from this transformation were used to overexpress the protein for purification. For this, cells were cultured at 37°C in LB medium containing 50 µg/ml kanamycin, 34 µg/ml chloramphenicol and 0.01% arabinose. Recombinant protein expression was subsequently induced by addition of IPTG (500 µM) at OD 0.65 together with supplementation of selenite (5 µM) and L-cysteine (100 µg/ml) and the cells were grown for an additional 16 h at room temperature for production. The overexpressed mutant protein was subsequently purified on a 2′,5′-ADP sepharose column (GE) from the cleared supernatant, obtained after lysozyme treatment (400 µg/ml) and centrifugation, essentially following the procedure described elsewhere [43], [44], [49] and above for the other TrxR preparations.

After production and purification as described above, all rat and human full-length or recombinant TrxR and SecTRAP preparations were subjected to desalting with NAP-5 columns (GE) for buffer change to 50 mM Tris-Cl, 2 mM EDTA, pH 7.5 (TE-buffer) and were kept in −20°C at a concentration of approximately 1 mg/ml until use. Protein concentrations were determined through the absorbance at 463 nm taking into account an extinction coefficient of 11300 M^−1^cm^−1^ for the FAD prosthetic group (which is present in both TrxR and SecTRAPs).

#### Derivatization with cisplatin

The rat C59S/C64S mutant TrxR, or recombinant rat TrxR as control for derivatization, were reduced with 0.5 mM DTT 20 min in room temperature before addition of CDDP (200 µM). The samples were then incubated for another 30 min, whereupon TrxR activity was measured in both samples using the direct DTNB assay [50]. Complete inhibition of the recombinant TrxR control sample was thereby confirmed and used as an indication that conditions for full derivatization of the Sec residue had been accomplished. Subsequently both protein preparations were desalted over a NAP-5 column equilibrated with TE-buffer and, to confirm irreversible derivatization of the Sec residue with CDDP, an additional DTNB assay was performed after the desalting step using the recombinant TrxR1 sample as control, which showed the expected lack of activity. Concentrations of these protein samples were determined by the Bradford method according to the protocol from BioRad using BSA as standard.

### Enzyme activity determinations

#### Determination of kinetic parameters

NADPH consumption measurements were performed by monitoring the change of absorbance at A_340_ for 20 min using either a Hitachi-557 or Schimadzu double beam spectrophotometer, essentially according to protocols described previously [27], [37], [51], [52]. Kinetic parameters for juglone were determined as described earlier [52]. The direct DTNB assay as a measure for mammalian TrxR1 activity was carried out as described [50] and parameters for the insulin assay are described in Table 2.

**Table 2:** No inhibition of TrxR1 activity *in vitro* by addition of SecTRAPs

Ratio between TrxR1:SecTRAPs a,	Insulin assay b,	TrxR1 activity DTNB assay c,
TrxR1 only	2048±317	2416±125
10∶1	2664±941	2418±66
1∶1	2702±532	2512±141
1∶10	2676±868	2524±90
1∶50	2409±211	3207±248
1∶2000^d^,	2265±247 d,	n.d.

The values are mean±S.D. of six different measurements

a, The TrxR1 preparation in this experiments was full-length rat TrxR1 and the SecTRAP preparation rat truncated TrxR1.

b, TrxR1 activity is given as turnover (min^−1^) calculated from the decrease at 340 nm detected in a microplate reader assay with 5 nM TrxR1, 0.5 nM-500 nM truncated TrxR1 as SecTRAP, 10 µM Trx, 145 µM Insulin in 50 mM Tris-Cl, 2 mM EDTA, pH 7.4 and 200 µM NADPH.

c, TrxR1 activity calculated from the increase at 412 nm detected in a microplate reader assay with 5 nM TrxR1, 2.5 mM DTNB in 50 mM Tris-Cl, 2 mM EDTA, pH 7.4 and 200 µM NADPH based upon comparisons to standard curves performed in normal quartz cuvettes with 1 cm light path length.

d, TrxR1 activity in insulin assay with 1∶10 molar ratio of Trx1 (1 µM) and truncated TrxR1 as SecTRAP (10 µM). The turnover (min^−1^) is calculated from the decrease at 340 nm detected in a microplate reader assay using 5 nM TrxR1, 145 µM insulin in 50 mM Tris-Cl, 2 mM EDTA, pH 7.4 and 200 µM NADPH.

n.d.: not determined

#### Stopped-flow kinetics

The rapid reaction studies using either truncated rat TrxR1 or full-length rat TrxR1 were performed under aerobic conditions using a DX.17MV stopped-flow spectrophotometer (Applied Photophysics) in 0.1 M K-phosphate buffer solution (pH 7.0), containing 1 mM EDTA, at 25°C, essentially following the procedures described previously [52]. The data were analyzed according to the single-exponential fit.

### Evaluation of a possible interaction between TrxR1 and SecTRAP in vitro

Rat TrxR1 (5 nM), human wild-type Trx1 (10 µM), kindly provided by Arne Holmgren, Karolinska Institutet, and NADPH (200 µM) were incubated in TE buffer containing 1 mg/ml BSA with different concentrations of truncated rat TrxR1 as a SecTRAP preparation, added as indicated in the text. Direct NADPH consumption was first assessed at A_340_ for 20 min (found to be negligible), whereupon insulin (145 µM) was added. The decrease at A_340_ was then followed for additional 30 min and NADPH consumption was calculated from the slope of the initial linear part of the curves. In addition, a similar assay was performed using DTNB as substrate, instead of Trx and insulin. For this, TrxR1 (20 nM), NADPH (0.8 mM) and varying concentrations of SecTRAPs where mixed in a final volume of 50 µl TE-buffer containing 1 mg/ml BSA in microtiter plate wells and then incubated 15 min at 37°C. Subsequently, 150 µl of a reaction mixture (2.7 mM EDTA, 67 mM Tris-Cl pH 7.4, 3.3 mM DTNB and 270 µM NADPH) was added and the increase of absorbance at A_412_ was followed for 3 min.

### Additional enzyme activity measurements

The NADPH consumption with subsequent estimation of free thiols was performed in 200 µl reaction mixes using a 96-well plate reader. In this assay, 10 nM of full-length or truncated rat TrxR1 was incubated with or without 10 µM human wild-type Trx1 and 145 µM insulin as indicated in the text, in 250 µM NADPH, 1 mg/ml BSA, 2 mM EDTA and 50 mM Tris-Cl (pH 7.5). Juglone was dissolved in DMSO and used at the indicated concentrations. Upon simultaneous addition of NADPH and mixing using a multipipette, NADPH consumption was followed at 340 nm over 30 min. Subsequently, 40 µl of each reaction was used for estimation of free thiols, stopping the reaction by addition of 160 µl 7 M GuHCl with 1 mM DTNB. Free thiol groups were determined using absorbance at 412 nm and an extinction coefficient of 13600 M^−1^cm^−1^. For determination of superoxide formation the adrenochrome method was used, based upon reduction of epinephrine by superoxide which can be quantified at 480 nm using an extinction coefficient of 4020 M^−1^cm^−1^, as described previously for studies of dinitrophenyl-derivatized TrxR [51]. Here the adrenochrome assay was performed in a 96-well plate reader with dual wavelength scan for the concomitant determination of NADPH consumption (using 340 nm and an extinction coefficient of 6200 M^−1^cm^−1^). For analysis whether the *BioPORTER* reagent itself may be reduced or redox cycle with TrxR or SecTRAPs, a reaction mixture containing 16 nM TrxR or truncated TrxR1 as SecTRAP, respectively, was made in complex with *BioPORTER* in the same ratios as in the regular cell experiments. These protein mixtures where then assayed, in the presence or absence of 5 µM juglone as positive control, in 300 µM NADPH, 2 mM EDTA and 50 mM Tris-Cl (pH 7.5) and NADPH consumption was monitored at 340 nm for 15 min. Potential superoxide production was also assessed using the adrenochrome method (see above for details of this method).

### Cell cultures and BioPORTER experiments

HEK293 (human embryonal kidney) cells (ATCC nr: CRL-1573) were grown in RPMI whereas A549 (human lung carcinoma) cells (ATCC nr: CCL-185) or HeLa (human cervical cancer) cells (ATCC nr: CCL-2) were grown in Dulbeccos modified Eagle medium with high or low glucose content, respectively. All media were supplemented with 10% heat-inactivated fetal calf serum, 2 mM L-glutamine, 100 units/ml penicillin and 100 µg/ml streptomycin and the cells were cultured in a humidified atmosphere of 5% CO_2_ at 37°C. For the different treatments, cells were seeded in LabTecII chamber slides (0.7 cm^2^/well) at a density of 10,000 cells per well 16 h before addition of protein/*BioPORTER*-complex, prepared according to the manufacturers protocol and shortly described as follows. An amount of 0.4 µg (for A549 or HEK293) or 0.2 µg (for HeLa) TrxR1 or SecTRAPs preparations (as described in the text) in TE-buffer (2 mM EDTA, 50 mM Tris-Cl, pH 7.5) was diluted in PBS to a final volume of 20 µl subsequently added to a “quick easy” *BioPORTER* tube (Gene therapy systems) by pipetting up and down 10 times to hydrate the dried compound. The protein/*BioPORTER* preparation was then incubated for 5 min in room temperature, briefly and gently vortexed for 5 s, whereupon it was mixed with 390 µl of serum-free medium. Subsequently, the mixture of 100 µl (for A549 or HEK293) or 200 µl (for HeLa) protein*/BioPORTER*-complex and medium (typically corresponding to a total amount of 100 ng TrxR or SecTRAP) was added to each well, containing cells that had first been washed with serum-free medium. To A549 and HEK293 cells, additional serum-free medium (100 µl) was finally added, resulting in a total volume of 200 µl in each well, that had been seeded with 10,000 cells 16 h prior to the experiment (see above). For assessment of concentration dependence, TrxR1 or SecTRAP was first diluted in TE-buffer containing 1 mg/ml BSA in a volume so that the resulting amount per well ranged from 1 pg to 100 ng TrxR1 or SecTRAP as stated in the text, always added to each well in the *BioPORTER* mix together with 100 ng BSA. In the experiments designed to assess possible protection from excess full-length TrxR1, 10 ng SecTRAPs per well were added together with the indicated amounts of TrxR1, ranging from 1 ng to 100 ng.

In the experiments with antioxidants, A549 cells were pretreated 1 h with 100 µM of ascorbic acid, 100 µM α-tocopherol, or a combination of both, in serum-containing medium. Fresh antioxidants (same concentrations) were subsequently added with the serum-free medium to the cells together with the protein/*BioPORTER*-complex. Controls were always made with TE-buffer, *BioPORTER* in absence of protein, SecTRAPs in absence of *BioPORTER*, or *BioPORTER* with only BSA, as indicated in the text.

In all cell experiments, C-terminally two-amino acid-truncated rat TrxR1 expressed as such in *E. coli* and purified over 2′,5′-ADP sepharose (see above) was utilized as SecTRAP preparation, unless stated otherwise.

### Cell viability assessment

The extent of cell death was determined with assessment through microscopy of fluorescent staining and morphology as described previously [37]. Shortly, cells were washed once with PBS after treatment for the indicated time with the *BioPORTER* preparations (4 h unless stated otherwise). Subsequently 100 µl of 10 µg/ml Hoechst 33342 in PBS was added to each well for blue staining of all nuclei. After 15 min incubation at room temperature 1 µl PI was added to a final concentration of 50 µg/ml for red staining of nuclei in (dead or dying) cells having defective and permeable membranes. Cells were then incubated for additional 5 min, whereupon they were washed once with PBS. Using a Hamamatsu digital camera 4742-95 with Leica DMRB microscope, three pictures were taken of the same field at 20-fold magnification using either white light or adapted with filters for Hoechst fluorescence (excitation: 360 nm, emission: 460 nm) or PI fluorescence (excitation: 570 nM, emission: 610 nm). Cell death was subsequently evaluated with assessment of the digital pictures, evaluating staining of the nuclei of a total of 700–1000 cells per well. Cells with blue-stained and PI-negative normal looking non-condensed nuclei were considered viable, whereas blue-stained cells with condensed nuclei or blue- as well as red-stained cells were considered dying and/or dead, as described previously [37] and also illustrated herein.

### Assessment of phosphatidyl serine exposure using Annexin-V staining

HeLa cells were seeded as described above and *BioPORTER* alone, SecTRAP/*BioPORTER*-complex or 1 µM staurosporine was added to the wells. After 3 h incubation, the cells were washed once with cold PBS and then once more with 1x Annexin-V binding solution, according to the protocol from the manufacturer (BD Biosciences). Subsequently 150 µl of 1x Annexin-V antibodies in binding solution were added to each well, followed by 15 min incubation. After addition of 1.5 µl of PI (1 mg/ml) the cells were incubated for additional 5 min, washed in Annexin-V binding buffer, and positive Annexin-V staining was subsequently visualized by fluorescence microscopy equipped with a filter for fluorescein isothiocyanate (excitation: 490 nm, emission: 525 nm), whereas any possible PI staining was assessed with the filter for texas red (excitation: 570 nm, emission: 610 nm).

### Analysis of caspase involvement

Cells were first seeded and grown as described above. For caspase inhibition experiments, the cells were preincubated 30 min with the indicated inhibitors at concentrations and conditions described in the text. Before treatment with protein/*BioPORTER*-complex the cells were then washed once with serum-free medium and new caspase inhibitor at the same concentration as during the preincubation was added together with protein/*BioPORTER*-complex in serum-free medium to the wells. Control cells were treated in the same manner with preincubation in serum-free medium, but with omission of caspase inhibitor. For the incubations using an extended duration of preincubation, serum-containing medium (final FCS concentration 10%) was added after 3 h. At the indicated time points, cell viability was evaluated as described above.

### Experiment with cycloheximide

In order to analyze the requirement of protein synthesis for cell death to occur, cells were seeded in LabTecII chamber slides (10,000 cells per well) and incubated over night whereupon they were pretreated 12 h with either cycloheximide (10 µM), TNF-α (10 ng/ml), the combination of cycloheximide (10 µM) and TNFα (10 ng/ml), or with only PBS as control, in all cases using medium supplemented with 10% FCS. Subsequently the cells were washed with serum-free medium before SecTRAP/*BioPORTER*-complex was added and incubation was continued for 4 h, followed by cell viability evaluation as described above.

### Detection of ROS production

HeLa cells were seeded in LabTecII chamber slides (10,000 cells per well) and incubated for 16 h. Some cells were pretreated 1 h with 100 µM α-tocopherol, 100 µM ascorbic acid or 100 µM of both compounds before addition of 100 ng of different protein*/BioPORTER*-complexes as described above and indicated in the figure legend. After 3 h incubation, cells were washed once in PBS before 15 min incubation with 2 µM DCFH and 10 ug/ml of Hoechst 33342. Cells were subsequently washed three times in PBS before ROS production was detected by confocal microscopy using filters for FITC, DAPI and rhodamine. All photographs of DCF fluorescence at a certain magnification but with different treatments were taken under identical incubation, excitation, and exposure conditions.

### Statistical analyses

Statistical analyses were performed with Prism 4 from GaphPad Software, using one-way ANOVA and the Tukey-Kramer test for determination of P values. The same software was used to draw graphs of the analyzed data.

## Results

### Human and rat SecTRAPs induce cell death in human A549 and HeLa cells

**Figure 1:**
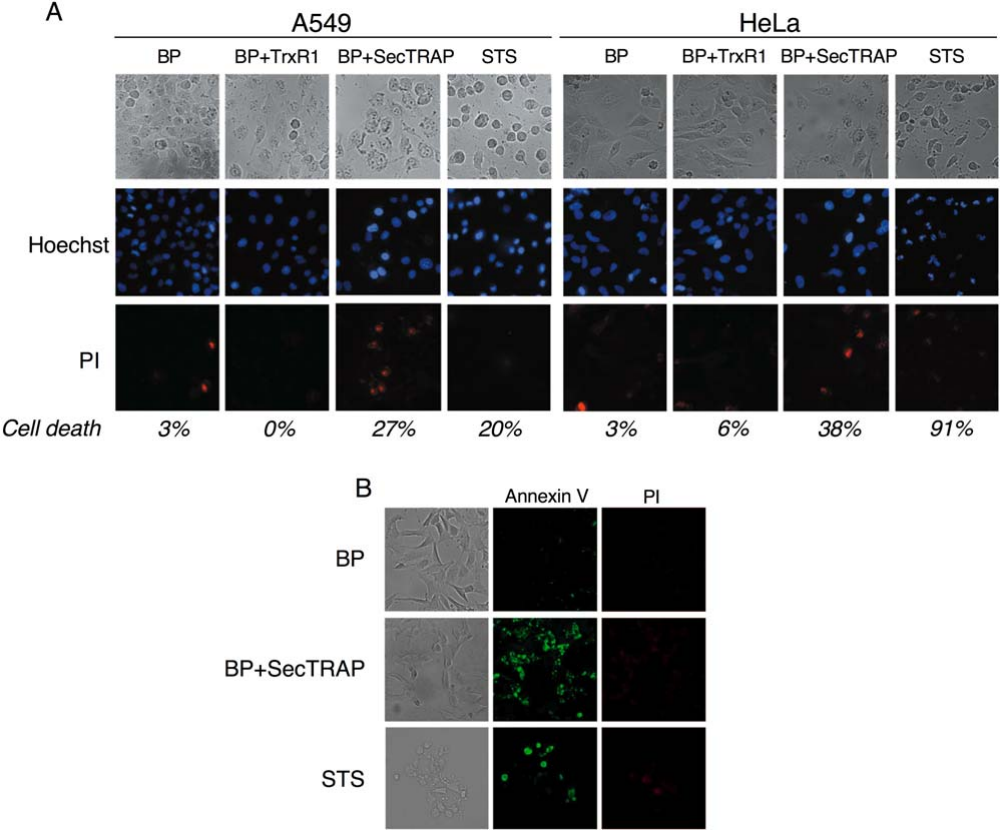
SecTRAPs induce cell death and phosphatidyl serine exposure in human cancer cells. (*A*) Morphological features and staining of HeLa and A549 cells after incubation for 4 h with *BioPORTER* (BP) alone, 100 ng full-length TrxR1/*BioPORTER-*complex, 100 ng SecTRAP/*BioPORTER-*complex or 1 µM staurosporine (STS), as indicated. Hoechst 33342 was used to visualize the shape or condensation of the nuclei and PI was used as a probe for lack of membrane integrity. Assessment of cell death was performed as described in the text. The percentage of cells denoted as dead, counting a total of 700–1000 cells in this particular experiment, is also given in italics in the lower part of the figure. (*B*) Exposure of phospatidylserine was evaluated after 3 h treatment of HeLa cells with either only *BioPORTER*, SecTRAP/*BioPORTER-*complex or 1 µM staurosporine, staining cells with Annexin-V and PI as described in the text. Magnification was x 40 in all panels.

The aim of this study was to analyze the characteristic features of SecTRAPs and to understand more about the cellular death mechanisms triggered by these proteins. The previously observed morphology of cells treated with SecTRAPs, showing PI uptake and condensed DNA [37], was not enough to discriminate between apoptosis and necrosis. Here we first confirmed that nuclear condensation and PI uptake was triggered by SecTRAPs in both A549 and HeLa cells, but not by full-length TrxR1, using protein delivery into the cells with *BioPORTER* (Fig. 1A). When treating HeLa cells with either SecTRAPs or staurosporine for only 3 h, we observed phosphatidyl serine exposure as demonstrated with Annexin-V staining. This apoptotic feature was detected prior to major uptake of PI at this early time-point, indicating that the cell membranes were still intact. The Annexin-V staining upon treatment with SecTRAPs was stronger than after treatment with 1 µM staurosporine, whereas no staining was observed with only *BioPORTER* (Fig. 1B) ruling out the possibility that the reagent as such affected the staining pattern. However, the morphology of the cells treated with 1 µM staurosporine as a positive control furthermore displayed cell membrane blebbing, which was not seen after SecTRAP treatment (Fig. 1B).

**Figure 2 pone-0001846-g002:**
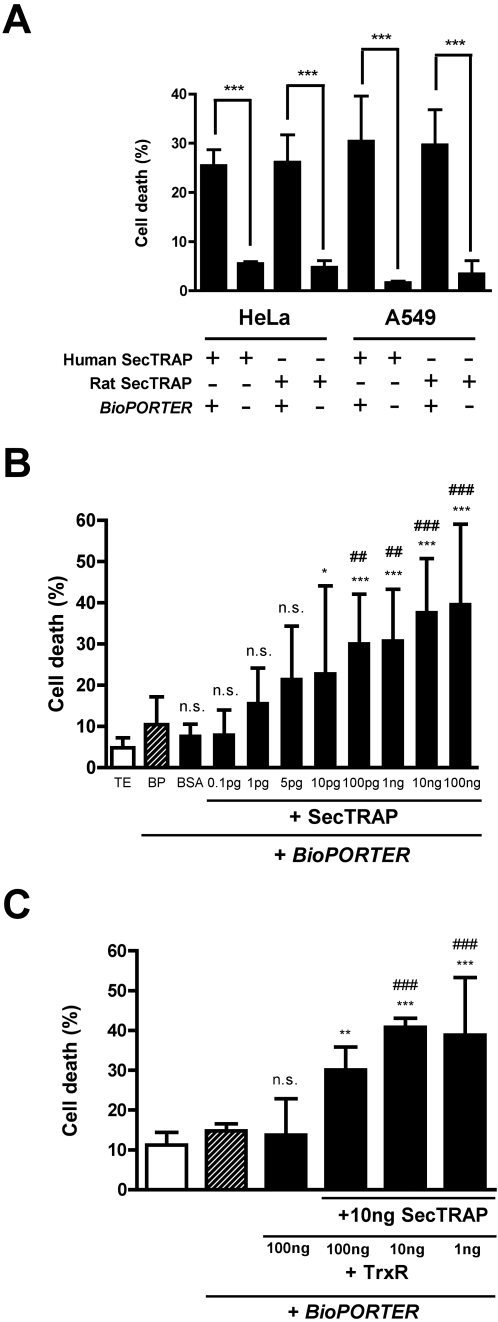
Concentration dependent cell death induction by SecTRAPs in two human cancer cell lines and the effects of excess TrxR1. (*A*) HeLa and A549 cells were treated with 100 ng rat or human SecTRAPs in the presence or absence of *BioPORTER* delivery reagent, as indicated in the figure and described in the text. A significant increase in cell death was seen in all cases where SecTRAPs were incubated with the cells in the presence of *BioPORTER* as compared to addition of SecTRAPs alone (***, p<0.001) (*B*) A SecTRAP preparation (truncated rat TrxR1) at an amount of 0.1 pg-100 ng was used for delivery into 10,000 A549 cells using *BioPORTER,* as described in the text. The graph shows the determined cell death (mean value±S.D.) triggered by each SecTRAP amount and significant differences to control treatments are indicated, using as controls either incubation with only TE buffer (white bar: n.s., p>0.05; *, p<0.05; **, p<0.01; ***, p<0.001) or with *BioPORTER* alone (dashed bar: n.s., p>0.05; ##, p<0.01; ###, p<0.001). No significant difference in cell death was seen comparing the two control treatments with each other. (*C*) A549 cells were treated with 100 ng full-length TrxR1 or a mixture of different amounts of TrxR1 with 10 ng SecTRAP using *BioPORTER*, as indicated in the figure. Differences in cell death were compared to control cells either treated with TE buffer (white bar; **, p<0.01; ***, p<0.001) or with only *BioPORTER* (dashed bar; ###, p<0.001). No statistically significant difference in cell death was seen between the two control treatments or in comparisons of either control with the treatment using 100 ng TrxR1 (n.s., p>0.05). In all experiments (*A–C*) cells were incubated for 4 h with the separate treatments and were subsequently stained with Hoechst 33342 and PI for evaluation of dead cells as described in the text.

In our previous study we introduced different preparations of recombinantly expressed rat TrxR1 to a human lung adenocarcinoma cell line (A549) [37]. Here we found that both human and rat SecTRAPs could effectively kill human HeLa and A549 cells (Fig. 2A). We could not, however, detect any increased cell death in HEK293 cells treated with SecTRAPs (not shown). We therefore decided to use the rat truncated TrxR1 protein as a SecTRAP preparation for our continued studies of cell death triggered in A549 and HeLa cells because of significantly higher yield in the recombinant expression system for rat TrxR1 than for human TrxR1, which is probably due to different rare-codon frequencies [43], [53], [54].

### Low amounts of SecTRAPs induce cell death and SecTRAPs are not direct inhibitors of the native thioredoxin system

The cell death-inducing effect of SecTRAPs was pronounced when 100 ng of protein was introduced to approximately 10,000 A549 or HeLa cells (Fig. 1 and Fig. 2A). A dilution series illustrated a clear trend towards an increase in cell death at treatment with as little as approximately 5 to 10 pg SecTRAP, but at least 100 pg was needed to give a statistically significant increased cytotoxicity using A549 cells treated for 4 h (Fig. 2B). With a SecTRAP mass of 55 kDa, 100 pg amounts to about 100,000 SecTRAP molecules per cell, thereby sufficient to provoke a significant increase of cell death in A549 cells, provided that all of the added molecules were indeed delivered into the cells. The killing effect was somewhat variable from experiment to experiment, but under the conditions utilized here about 30–40% dead or dying cells were typically observed after 4 h treatment with 100 pg SecTRAPs or more (Fig. 2B). In line with these findings, it should be noted that 10,000 A549 cells contain about 1–2 ng endogenous native TrxR1 and the results thereby suggest that the cell death provoked by SecTRAPs in susceptible cells can occur at rather low amounts, compared to the endogenous TrxR1 levels.

It was suggested that SecTRAPs could interact with enzymatically active Trx1 endogenously present in the cells and thereby inhibit Trx1-dependent reactions as part of the SecTRAP effect [55]. Here we wished to experimentally assess such potential interaction. For this, we first analyzed *in vitro* whether a 10-fold molar excess of SecTRAPs over Trx1 and 2000-fold excess over TrxR1 could inhibit the enzymatic activity of TrxR1 in a Trx1-linked insulin reduction assay. We also studied the effects of SecTRAPs in a direct TrxR1 activity assay using DTNB as substrate. In neither case could we find evidence of an inhibition of TrxR1 activity by an excess of SecTRAPs (Table 2). We then analyzed whether A549 cells could be protected from SecTRAP-induced cell death by the simultaneous addition of an excess of enzymatically active full-length TrxR1. Using co-delivery of 10 ng SecTRAP with 100 ng TrxR, i.e. a 10-fold excess of TrxR over SecTRAP protein, and about 50-fold excess of TrxR added over the endogenous cellular level of TrxR, this indeed counteracted the SecTRAP effect to some extent, while at equimolar amounts or less of TrxR compared to SecTRAP no protection was observed (Fig. 2C). These results (Table 2 and Fig. 2C) suggest that SecTRAPs can provoke a cell death via a mechanism not necessarily involving direct interactions with the endogenous proteins of the thioredoxin system, but that the thioredoxin system or at least TrxR1 may possibly have some protective effect if present in large excess compared to the SecTRAP level.

### The cell death provoked by SecTRAPs occurs without induction of novel protein synthesis

**Figure 3 pone-0001846-g003:**
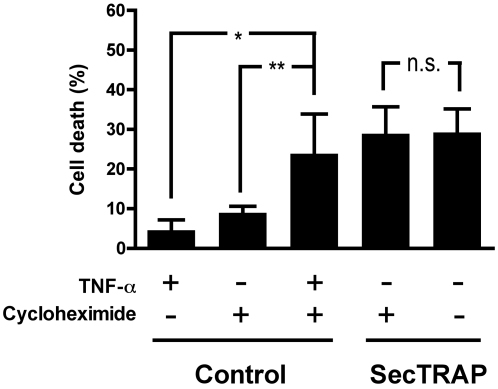
Cell death by SecTRAPs is not dependent upon induction of protein synthesis. HeLa cells were preincubated 12 h with TNF-α, cycloheximide or a combination of TNF-α and cycloheximide, whereupon SecTRAP/*BioPORTER-*complex was added as indicated and the cells were then incubated for additional 4 h. Cell death was subsequently evaluated by staining with Hoechst and PI as described. As a control experiment, an expected increase of cell death was seen when cells were treated with the combination of TNF-α and cycloheximide compared to treatment of either of these compounds alone, showing that the cycloheximide treatment had inhibited protein synthesis (see text). In contrast, cycloheximide had no effect on the cell death provoked by the SecTRAP/*BioPORTER* treatment, as indicated in the figure (n.s., p>0.05; *, p<0.05; **, p<0.01).

We next wished to study whether the cell death effect of SecTRAPs required induction of novel protein synthesis. For this, HeLa cells were preincubated with the protein synthesis inhibitor cycloheximide for 12 h before treatment with SecTRAPs. As a positive control for inhibition of protein synthesis, cycloheximide was co-incubated with TNFα, which is a combinatory treatment known to induce apoptosis in HeLa cells under these conditions [56]. As expected, treatment with either the combination of cycloheximide and TNFα, or with SecTRAPs alone, induced cell death in HeLa cells (Fig. 3). However, cycloheximide had no effect on the cell death provoked by SecTRAPs (Fig. 3), suggesting that novel protein synthesis was not required for the cytotoxicity.

### Caspase inhibitors can protect cells from SecTRAPs

**Figure 4:**
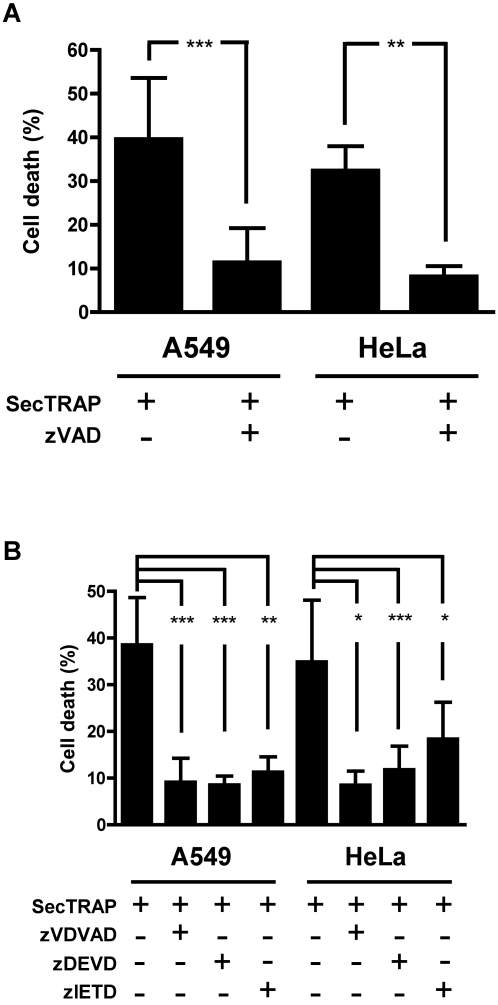
Cell death induction by SecTRAPs is prevented by caspase-2 and caspase-3/7 inhibitors. (*A*) shows that the cell death provoked by SecTRAPs is significantly decreased upon preincubation of either A549 or HeLa cells for 30 min with 100 µM of the general caspase inhibitor zVAD before the SecTRAP treatment (**, p<0.01; ***, p<0.001). In (*B*) HeLa or A549 cells were incubated 30 min with 25 µM of inhibitors for caspase-2 (zVDVAD-fmk), caspase-3 (zDEVD-fmk) or caspase-8 (zIETD-fmk) before SecTRAP treatment. In all of these cases a significantly lower cell death was observed, as indicated in the figure, suggesting that the three caspases may be involved in propagating the cell death triggered by SecTRAPs, as further discussed in the text (*, p<0.05; **, p<0.01; ***, p<0.001).

The pan caspase inhibitor zVAD-fmk was first used to assess whether the cell death provoked by SecTRAPs could be prevented by general caspase inhibition. Both A549 and HeLa cells were well protected against the effects of SecTRAPs when preincubated with a high concentration (100 µM) of the general zVAD-fmk caspase inhibitor (Fig. 4A). This result suggested that SecTRAPs may provoke a cell death with apoptotic features, but high concentrations of caspase inhibitor may also mask cell death by mitotic catastrophe. However, a lower concentration of zVAD (25 µM) was protective for up to 12 hours (not shown), as were more specific caspase inhibitors. With two initiator caspases, caspase-8 and caspase-2, converging in activation of caspase-3 [57], [58], we decided to focus on these three caspases. A caspase-3 inhibitor (zDEVD-fmk) at 25 µM almost completely prevented the cell death provoked by SecTRAPs in either HeLa or A549 cells (Fig. 4B). An inhibitor of caspase-8 (zIETD-fmk) at the same concentration reduced the apoptotic effects of SecTRAPs in HeLa cells to about half, while it was highly protective in A549 cells. This indicated that cell death upon exposure to SecTRAPs could involve caspase-8, but the notable apoptosis seen in HeLa cells also in presence of inhibitor suggested that an additional initiator caspase could be involved. Notably, the caspase-2 inhibitor zVDVAD-fmk effectively blocked the apoptosis provoked by SecTRAPs in both A549 and HeLa cells, to the same total extent as the inhibitor of caspase-3 (Fig. 4B). These results may indicate that both caspase-2 and caspase-3 could be involved in the cell death provoked by SecTRAPs.

### SecTRAPs are efficient reductases with juglone in a reaction dependent upon the N-terminal redox active disulfide/dithiol motif

In our previous study we showed that SecTRAPs display very low direct NADPH oxidase activity, i.e. in absence of other substrates than NADPH and oxygen, apart from the case when TrxR1 has been derivatized with dinitrohalobenzenes [37]. We also found that glutathione reductase, having higher inherent NADPH oxidase activity than the selenium-compromised forms of TrxR1, lacked the capacity to induce cell death [37]. We thus initially speculated that a direct prooxidant capacity of SecTRAPs should not be the reason for the cell death induction. However, we here hypothesized that SecTRAPs, still having a functional FAD moiety and an intact N-terminal redox active CVNVGC motif (see *Introduction*), could perhaps react with some endogenous cellular substrate or target as a part of the apoptotic mechanism. To test whether the N-terminal CVNVGC motif was important for the SecTRAP effect we therefore generated a mutant where the two cysteine residues in this motif were exchanged for serine moieties. This protein was made to maintain an intact C-terminal –GCUG motif and the incorporation of selenocysteine was verified by ^75^Se labeling (not shown). We first performed *in vitro* analysis of the NADPH consumption of the C59S/C64S mutant compared to other TrxR1 or SecTRAP preparations, using the quinone substrate juglone, which was previously studied with non-modified TrxR1 and found to be reduced by the N-terminal CVNVGC motif [27], [52]. As expected the NADPH consumption of the C59S/C64S mutant, either with the Sec residue intact or with this residue derivatized with cisplatin, was negligible. We concluded that the C59S/C64S mutant is, in principle, a completely redox inert form of modified TrxR1. In contrast, a SecTRAP preparation, here represented by the two-amino acid truncated TrxR1 (with an intact N-terminal CVNVGC motif), was found to efficiently reduce juglone, at a rate comparable to that of full-length TrxR1. This finding was in line with our earlier results, showing that juglone reduction can occur at the other redox active sites of TrxR1 than the Sec-containing selenolthiol motif [27], [52]. It should again be emphasized in the context of the present study that in spite of efficient juglone reduction, the SecTRAP preparation completely lacked Trx reducing activity. These results are summarized in Table 3.

**Table 3:** Steady state kinetic properties of TrxR1, SecTRAPs and the C59S/C64S mutant

Enzyme and substrate	*k*_cat_ (s^−1^)	*K*_m_ (µM)	*k*_cat_/*K*_m_ (µM^−1^s^−1^)
TrxR1 and juglone a,	5.5±0.2	2.4±0.3	2.3±0.1
SecTRAPs and juglone a,	6.5±0.6	4.4±1.1	1.4±0.3
C59S/C64S and juglone	0.4±0.01	15.5±2.2	0.02
TrxR1 and Trx b,	27.5	2.5	11
SecTRAPs and Trx	< 0.01 c,	n.a.	n.a.
C59S/C64S and Trx	< 0.01 c,	n.a.	n.a.

a, *k*_cat_ per active site and *K*_m_ were calculated by following the NADPH consumption as described in the text.

b, Values taken from [50] and adjusted to *k*_cat_ per active site.

c, Measured in the insulin assay, under the same conditions as given in Table 2.

n.a., not applicable.

**Figure 5:**
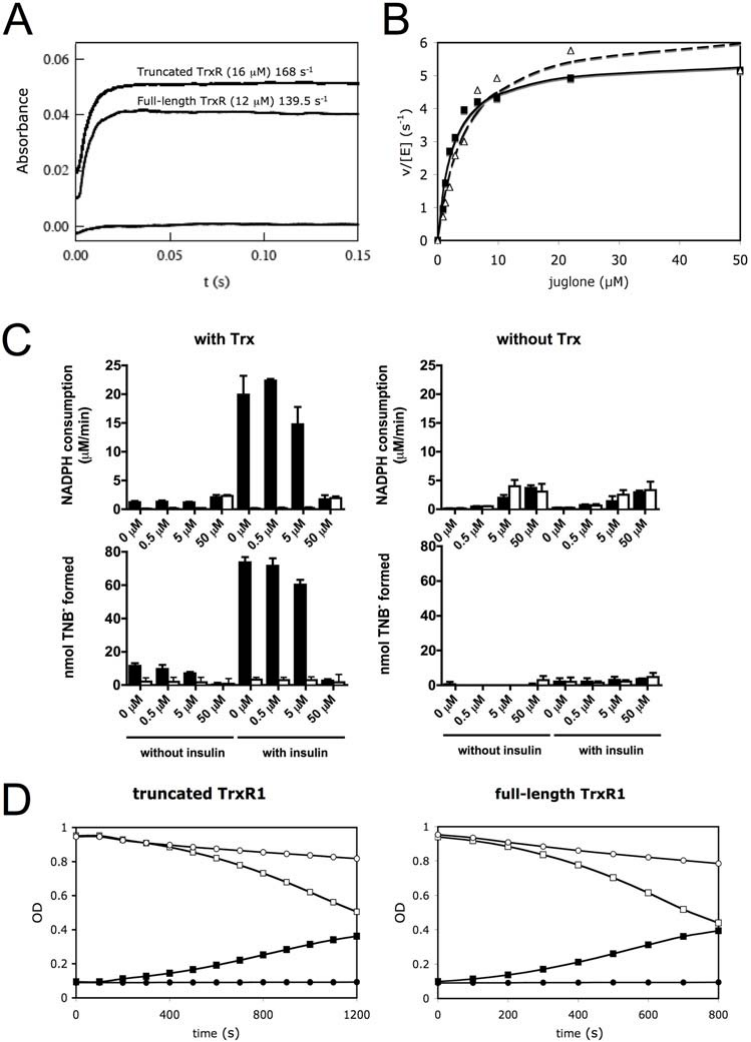
Both SecTRAPs and TrxR1 are efficient in reducing juglone and thereby produce superoxide. In (*A*) the formation of FAD-reduced disulfide charge transfer complex by NADPH (80 µM) in a SecTRAP preparation (truncated TrxR1, 16 µM subunit) and full-length TrxR1 (12 µM subunit) was analyzed with stopped-flow spectroscopy at 540 nm, showing similar kinetics for both enzymes. In (*B*) Michaelis-Menten kinetics for both full-length (filled symbols) and truncated (open symbols) TrxR1 using juglone as a substrate is demonstrated. In (C) it is shown that Trx1 and juglone compete for the reduction by full-length TrxR1 (filled bars) but that truncated TrxR1 (open bars) can only use juglone and not Trx1 as a substrate. This is illustrated from the initial NADPH consumption rate (0–200s) followed at 340 nm with or without Trx1 and insulin (upper panels). After 30 min of reaction, the number of exposed free thiols was determined (lower panels) in order to estimate to which extent the electrons from the NADPH oxidation were passed on to Trx1 and subsequently to insulin. The juglone concentration is indicated at the x-axes and each bar represents the mean±S.D. of three measurements. In (D), the reduction of juglone (5 µM) catalyzed by 10 nM SecTRAP (truncated TrxR1, left panel) or full-length TrxR1 (right panel) is shown following the consumption of NADPH (initial concentration 250 µM) by the decrease in absorbance at 340 nm (open symbols). Concomitantly, superoxide formation was detected at 480 nm with the adrenochrome method using 2 mM epinephrine (filled symbols). The formation of adrenochrome was completely inhibited by addition of 5 U SOD (circles), which also reduced the elevated NADPH consumption seen upon addition of only epinephrine (squares).

Analyzing the stopped-flow kinetics of truncated rat TrxR1 as a SecTRAP preparation as well as full-length rat TrxR1, we observed that NADPH reduced the FAD moiety of both proteins at similar rates. The SecTRAP preparation formed the FAD-thiolate charge-transfer complex typical of mammalian TrxR enzymes, having an absorbance maximum at 540 nm [19], [27], with a rate of ∼168 s^−1^ and the corresponding rate for full-length TrxR1 was ∼140 s^−1^ (Fig. 5A). In addition, juglone reduction, using lower concentrations under steady state conditions, displayed an acceleration during reactions with either of the two proteins, as found earlier for TrxR1 [27]. Apparent kinetic parameters in the juglone reduction for the initial ∼0–20 s time period were in the same range for both proteins (Fig. 5B) and determined here to *k*_cat_ ∼6 s^−1^ and *k*_cat_/*K*_m_∼1–2×10^6^ M^−1^s^−1^ (Table 3). These findings illustrate that SecTRAPs can propagate rapid redox reactions with selected substrates, although they lack the proper Sec-containing active site of TrxR1.

### The effects of thioredoxin on the oxidoreductase activity of SecTRAPs using juglone

Both Trx1 and juglone can compete as substrates for reduction by full-length TrxR1. SecTRAPs however, can only reduce juglone and not Trx1 (Table 3). We therefore wished to analyze whether reduction of juglone by SecTRAPs could occur also in the presence of Trx1, which would possibly suggest that SecTRAPs can react with alternative substrates also in a cellular context where Trx1 would be present. We found that Trx1 at a physiological concentration (10 µM) could lower the juglone reduction by truncated TrxR1 using low concentrations of juglone (5 µM or less). However, 10 µM Trx1 had no effect on the reduction of 50 µM juglone by truncated TrxR1, as demonstrated by the same NADPH oxidation rate either in the presence or absence of Trx1 and its disulfide substrate insulin (Fig. 5C, upper panels, white bars). It should be noted that this activity was dependent upon direct juglone reduction by the SecTRAP protein, since this truncated enzyme has no activity with Trx1. This fact was also illustrated by the lack of Trx-coupled insulin reduction in the assay (Fig. 5C, lower left panel, white bars). The properties of the SecTRAP protein were in sharp contrast to those of full-length TrxR1, which has a preference for Trx1 as substrate, being essentially unaffected by addition of low concentrations of juglone in the presence of Trx1 and insulin. This was clearly illustrated by the channeling of electrons from NADPH (Fig. 5C, upper left panel, black bars) to production of free insulin-derived thiols, formed through insulin disulfide reduction by Trx1 in the reaction propelled by the TrxR1 enzyme (Fig. 5C, lower left panel, black bars). However, the Trx1-reducing activity of full-length TrxR1 was noticeably impaired at the higher concentration of juglone (50 µM) through the effects of juglone as inhibitor and a subversive substrate for the enzyme [27]. These results collectively suggested to us that TrxR1 preferentially reduces Trx1 when present, whereas SecTRAPs albeit not being able to reduce Trx1, can still be active as oxidoreductases able to reduce other substrates than Trx1. As we found next, this activity can confer notable prooxidant properties, that also correlate to the cytotoxic effects of SecTRAPs.

### Superoxide is produced during reations with juglone

We detected superoxide production during juglone reduction, by either SecTRAPs or TrxR1, as assessed with the adrenochrome method. Interestingly, addition of epinephrine also increased the rate of NADPH consumption (Fig. 5D). Addition of excess SOD completely abolished the adrenochrome formation, confirming that superoxide was formed (Fig. 5D). These findings were possibly suggestive of one-electron reduction of juglone, producing superoxide, which would be in contrast to the predominant two-electron reduction mechanism found earlier using 1,4-benzoquinone as a quinone substrate for TrxR1 [27]. As reported in our earlier study, TrxR1 is an efficient reductase for several quinones in addition to juglone, i.e. in the absence of Trx1, including 9,10-phenanthrene quinone [27]. The latter substrate is reduced solely by the Sec-containing C-terminal active site [27]. Accordingly, we found here that SecTRAPs are completely inactive in reduction of 9,10-phenanthrene quinone (not shown), in contrast to their efficient reduction of other substrates such as juglone.

### The cell death provoked by SecTRAPs correlates to the prooxidant capacity

**Figure 6:**
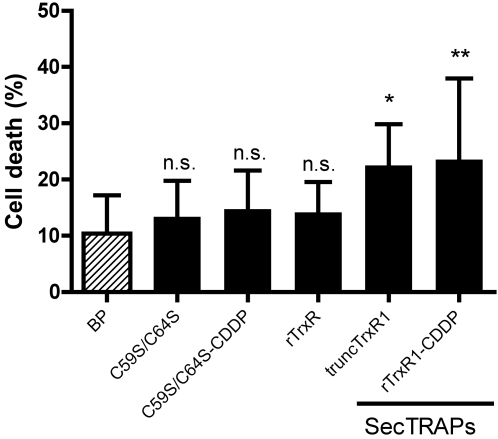
C59S/C64S mutant SecTRAPs cannot induce cell death in A549 cells. A549 cells were treated with 100 ng of the C59S/C64S mutant rat TrxR or native rat TrxR1 preparations, with or without cisplatin (CDDP) derivatization of the Sec residue, using *BioPORTER,* as indicated in the figure and described further in the text. Cell death was significantly increased after treatment with truncated TrxR or TrxR1 derivatized with cisplatin compared to control using only *BioPORTER,* whereas the other proteins gave no significant difference in cell death compared to the control (n.s., p>0.05; *, p<0.05; **, p<0.01).

When the different TrxR1 or SecTRAP preparations that we had analyzed for juglone reduction (Table 3) were introduced into A549 cells, we found that the C59S/C64S mutants could not provoke cell death at any higher extent than non-compromised TrxR1 or the control using only *BioPORTER* treatment, in contrast to the cell-killing SecTRAP capacity of either TrxR1 that had been derivatized with cisplatin, or of truncated TrxR1 (Fig. 6). This showed that an intact CVNVGC motif of the selenium-compromised forms of TrxR1 was required for SecTRAP properties. Considering the different enzymatic activities of the pure protein preparations (Table 3, Fig. 5) in line with their cell killing capacity (Fig. 6), it became apparent that only those TrxR1-derivatives having the capacity to support efficient reduction of juglone and at the same time lacking Trx1 reducing activity, were functional as SecTRAPs. This suggested to us that in a cellular environment, non-derivatized native TrxR1, albeit having quinone reductase activity *in vitro* as both found here (Table 3, Fig. 5) and previously shown [27], may easily be saturated by the endogenous Trx1 substrate and thus mainly propagate Trx1-dependent cellular functions. SecTRAPs, in contrast, may kill cells due to their predominant prooxidant capacity (Fig. 5D), provided that there is a cellular substrate that could take the place of juglone used in our *in vitro* assays.

**Figure 7 pone-0001846-g007:**
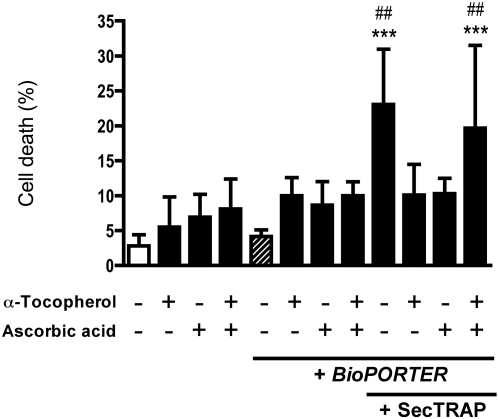
Either α-tocopherol or ascorbic acid can prevent cell killing by SecTRAPs but not the combinatory treatment. A549 cells were preincubated for 1 h with α-tocopherol (100 µM), ascorbic acid (100 µM) or a combination of the two compounds, as indicated in the figure. SecTRAP/*BioPORTER*-complex was subsequently added to the cells, which were then incubated for additional 4 h before analysis of cell death as described in the text. A significant increase in cell death compared to non-treated cells (***, p<0.001) or cells treated with only *BioPORTER* (##, p<0.01, ###, p<0.001) was seen in cells treated with SecTRAPs either in absence of the antioxidant compounds or together with the combination of both α-tocopherol and ascorbic acid. All other treatments lacked a significant difference in cell death compared to either of the two controls (p>0.05).

Pretreatment with antioxidants would likely protect cells against the toxic effects of SecTRAPs if a prooxidant effect is the mechanism for cell death induction. To test this, we preincubated cells with either water-soluble ascorbic acid (Vit C), lipid soluble α-tocopherol (Vit E), or a combination of both, with subsequent assessment of the induction of cell death by SecTRAPs. The background cell death with only antioxidant treatment was 5–10% and increased marginally to 10–15% together with *BioPORTER*. Upon treatment with SecTRAPs in the absence of antioxidants the cell death reached the same level as seen previously, but this effect could indeed be blocked by either antioxidant alone (Fig. 7). Interestingly, however, the combinatory treatment of both antioxidants together with the SecTRAP/*BioPORTER-*complex resulted in a similar level of cell death as that seen with SecTRAP/*BioPORTER* alone, while the combination of both antioxidants together with only *BioPORTER* but without SecTRAP, had no such effect (Fig. 7). These results suggested that antioxidant treatment can indeed prevent SecTRAP-provoked cell death, but also that SecTRAPs can propagate a cytotoxicity derived from the simultaneous treatment with ascorbate and α-tocopherol.

**Figure 8:**
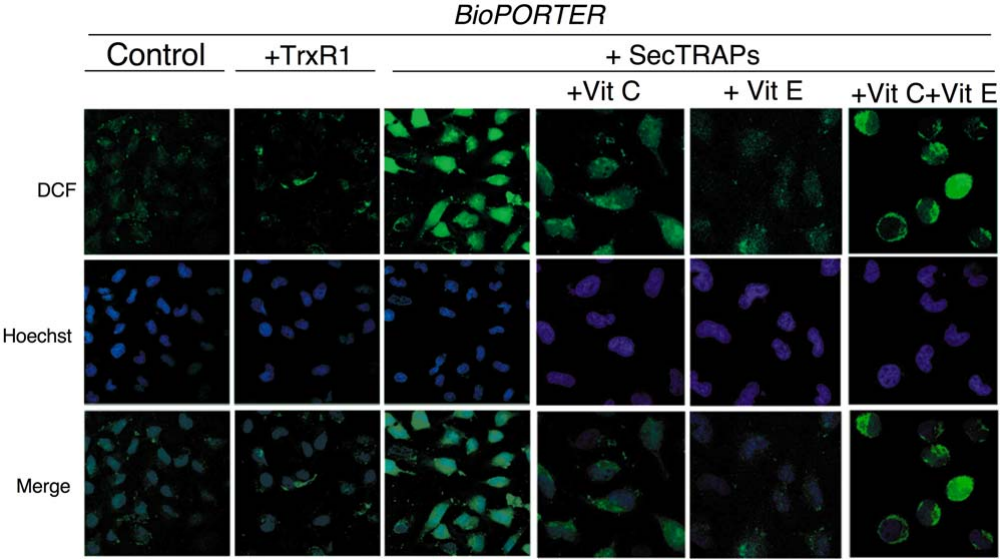
HeLa cells treated with SecTRAPs show an increased production of reactive oxygen species that was quenched by either α-tocopherol or ascorbic acid but not by the combinatory treatment. HeLa cells were treated with *BioPORTER*, TrxR1/*BioPORTER* or SecTRAPs/*BioPORTER* for 3 h as indicated in the figure, whereupon the images of the cells stained with the ROS-sensitive marker DCFH were taken, as described in the text. The cell nuclei were counterstained with Hoechst 33342. Ascorbic acid (Vit C) and/or α-tocopherol (Vit E) was added 1 h in advance to the cells before treatment. Two distinct experiments with two samples in each treatment were performed with similar results and representative images of the observed staining patterns are shown. The three right-most pictures displaying the intracellular patterns of DCF fluorescence are shown at higher magnification than the three panels to the left displaying the overview of DCF fluorescence in control cells or in cells treated with either TrxR1 or SecTRAPs together with *BioPORTER*.

To further analyze whether oxidative stress was indeed provoked in these experiments, we subsequently used DCFH as a probe in experiments treating HeLa cells with either TrxR1 or a SecTRAP preparation, in combination with the antioxidant compounds. Cells treated with SecTRAPs displayed an intensive green fluorescence of DCF, which was not observed after treatment with TrxR1 or *BioPORTER* alone (Fig. 8, left three panels). The DCF signal was quenched after treatment with either ascorbate or α-tocopherol, although DCF fluorescence was detectable in some cells after treatment with ascorbate (Fig. 8, right three panels). Interestingly, the combinatory treatment of both antioxidants together with SecTRAPs gave a strong DCF signal, yet in a different pattern compared to SecTRAPs alone (Fig. 8). This finding substantiated that SecTRAPs could propagate an oxidative stress, either directly in cells or in concert with the combination of ascorbate and α-tocopherol as used herein. The intensity of DCF fluorescence thus correlated well with the observed cell death using the same treatments (cf. Fig. 7 and Fig. 8).

## Discussion

Here we have found that cell death provoked by certain forms of selenium compromised mammalian TrxR1 (SecTRAPs) shows both apoptotic features, such as Annexin-V staining and protection by caspase inhibitors, as well as necrotic features, such as lack of membrane blebbing or nuclear segmentation and an extensive direct PI uptake, the latter which indicated that cellular membrane integrity was rapidly lost. Those findings in combination with the intracellular ROS production triggered by SecTRAPs, as shown by DCF fluorescence and the protection from cell death by antioxidants, collectively show that the selenoprotein TrxR1, a key player in antioxidant defense, can be converted into a potent prooxidant killer of cells when its highly reactive Sec residue becomes compromised.

The notion that SecTRAPs trigger cell death, at least in part, by a provoked oxidative stress is compatible with several earlier observations. It agrees well with the fact that DNCB as well as juglone are compounds that target and inhibit cellular TrxR1 with concurrent induction of a cell death, showing necrotic features but also involving caspase-3/7 activation and being strikingly different in properties compared to the cell death triggered by staurosporine [52]. Formation of SecTRAPs may possibly also be part of the mechanism for induction of apoptosis by the TrxR1-specific inhibitor auranofin in both cisplatin-sensitive and –resistant cancer cells, shown to be related to production of reactive oxygen species as well as the levels of thioredoxin reductase [59]. It was furthermore shown that targeting of TrxR1 by electrophilic prostaglandin derivatives induces a different type of cell death, than that resulting from siRNA-mediated knockdown of TrxR1 [36], [60]. In addition, derivatization of TrxR1 by cisplatin with formation of SecTRAPs may be one explanation for the ROS formation and triggering of apoptotic pathways in cytoplasts treated with cisplatin, where DNA damaging effects evidently cannot play a role [61]. SecTRAP formation could potentially be a factor contributing to the cytotoxic effects of several known TrxR1 inhibitors, such as curcumin [62], arsenic trioxide [30], 4-hydroxy-2-nonenal [63], or some of the additional TrxR1 inhibitors discussed elsewhere [6], [64]–[66]. It is noteworthy that TrxR1 inhibitors often result in more pronounced oxidative stress and cytotoxicity than knockdown of TrxR1 using siRNA. The latter seems to result in slower cell growth, but without clear signs of oxidative stress or apoptosis [67], [68] and, as recently shown, without evident oxidation of Trx1 [69]. In contrast, treatment of cells with one inhibitor of TrxR1 (monomethylarsonous acid) can lead to Trx1 oxidation and ROS formation whereas another inhibitor (aurothioglucose) did not show such effects, in spite of about 90% reduction in the total cellular TrxR activity in both cases [69]. Reasons for different effects between use of different TrxR1 inhibitors or in comparison to TrxR1 knockdown experiments could, naturally, include potential interactions of the inhibitors with additional cellular targets apart from TrxR1. Another explanation could however also be that formation of SecTRAPs occurs upon use of certain TrxR1 inhibitors, i.e. by those inhibitors specifically targeting the Sec residue to the enzyme. This can, as shown herein, result in a gain of function of the protein that can provoke oxidative stress and cell death, thereby not solely being the result of diminished TrxR1 activity.

We found earlier that glutathione reductase (GR), having higher inherent NADPH oxidase activity *in vitro* than SecTRAPs, could not provoke cell death [37]. The likely reason should be that the NADPH oxidase activity of GR is suppressed in a cellular context, where the enzyme would easily be saturated with its natural substrate glutathione. It should furthermore be noted that the protein surface charge distribution patterns of GR and TrxR1 are strikingly different from each other; this was suggested to explain the different substrate specificities between these two enzymes, even upon removal of the selenolthiol motif in TrxR1, although the two enzymes are otherwise closely related in overall domain configuration [70]. This difference may be part of the explanation why TrxR1, or SecTRAPs, are about one order of magnitude more efficient than GR in reduction of several quinone substrates [27].

Our findings that SecTRAPs can be potent prooxidant enzymes, although they completely lack the native Trx1 reducing capacity of TrxR1, could explain much of their toxic properties. We have not yet identified any cellular endogenous substrate(s) that interact with SecTRAPs. However, the findings with the model substrate juglone clearly demonstrated that SecTRAPs have the capacity to become potent superoxide-producing NADPH oxidases, also in the presence of physiological concentrations of Trx1, provided that intracellular substrates exist at sufficient concentration together with which SecTRAPs can propagate prooxidant reactions. The prominent oxidative stress seen upon treatment of cells with SecTRAPs, as illustrated by the increased DCF fluorescence and the protective effects of antioxidants, suggest that such intracellular substrates exist.

The observation that SecTRAPs could propagate intracellular ROS production and cell death upon the combinatory treatment with ascorbic acid and α-tocopherol, whereas either of the two compounds alone protected the cells, was an intriguing finding. It can likely be explained by the redox properties of these compounds in connection with the properties of SecTRAPs and the finding further supports our view that directly SecTRAPs propagate oxidative stress, coupled to some hitherto unidentified cellular substrate(s). It should be noted that antioxidant compounds, like ascorbate and α-tocopherol, all have the inherent capacity to act as prooxidants because they easily form radical compounds, with their cellular effects thereby being a matter of concentration and the microenvironment within which they act. If SecTRAPs promote the initiating oxidative event and ascorbic acid and α-tocopherol are present at high equal concentrations, this may potentially first lead to the formation of ascorbyl radicals, which in turn may react with α-tocopherol to form tocopheryl radicals. Thereupon the electrons can be transferred to subsequent cellular compounds, induce propagation of radical formation and give rise to the observed oxidative stress. This potential reaction, from SecTRAPs over ascorbate to tocopheryl radical formation, should be amplified when α-tocopherol is added concomitantly with ascorbic acid and SecTRAPs. If ascorbic acid alone is instead added in excess, two ascorbyl radicals that are formed through the prooxidant effects of SecTRAPs, could easily dismutate into one ascorbate and one dehydroascorbate molecule, thereby acting as chain-breaking antioxidant. If α-tocopherol alone is added in excess, this may potentially protect cellular lipids from oxidative damage, but SecTRAPs are probably less likely to induce tocopheryl radicals by direct interactions, while in contrast a direct reaction with ascorbic acid is indeed plausible. Interestingly, dehydroascorbate formed from the ascorbyl radical dismutation can be reduced back to ascorbic acid by TrxR1. For further discussions on the web of interactions and reactions between TrxR1, ascorbic acid and α-tocopherol, see an earlier review on the subject [8].

In this study, we used a two-amino acid C-terminally truncated form of TrxR1 as the major model protein for studies of SecTRAPs. Such truncated TrxR1 could perhaps be produced in certain cells during translation by a truncation at the Sec-encoding UGA codon. However, the interplay between UGA-directed Sec insertion and translational termination is far from fully understood in mammalian cells and the UGA-truncated form of TrxR1 has not yet been conclusively demonstrated in analyses of selenium-starved tissue. Only direct demonstration of the two-amino acid truncated TrxR1 species purified from selenium-depleted tissues would answer whether such protein species may be formed under natural conditions of selenium depletion. To our knowledge, no studies have yet been published demonstrating endogenous production of the truncated enzyme in mammalian tissues. Evidence for production of a truncated TrxR1 species was reported for NCI-H441 cells [71], while in other liver or kidney-derived cell lines clear evidence for such production at selenium depletion could not be seen [72]. The toxicity of SecTRAPs to A549 and HeLa cells could possibly explain prior difficulties encountered in attempts to establish a stable TrxR1-overexpressing cell line. After several unsuccessful attempts in different tumor cell lines (Jurkat, HeLa, U1285), an overproducing stable transfection was finally achieved in HEK293 cells, as reported elsewhere [73], which is a cell line that we found to be resistant to SecTRAPs. It is possible that the difficulty to stably overexpress TrxR in several different cell lines, may have been due to the fact that under such overproducing conditions, UGA-truncated forms of TrxR1 (i.e. SecTRAPs) are formed, while the HEK293 cells in which the overproduction was successful are resistant to SecTRAPs by a mechanism yet to be elucidated. Although it is not clear whether cells or tissues may become exposed to truncated TrxR1 under certain cases of selenium limitation, formation of SecTRAPs can nonetheless occur through the direct derivatization of the Sec residue of TrxR1 by electrophilic compounds, either endogenously produced in cells, exposed in the form of xenobiotics or environmental contaminants, or purposely given in the form of alkylating drugs.

**Figure 9:**
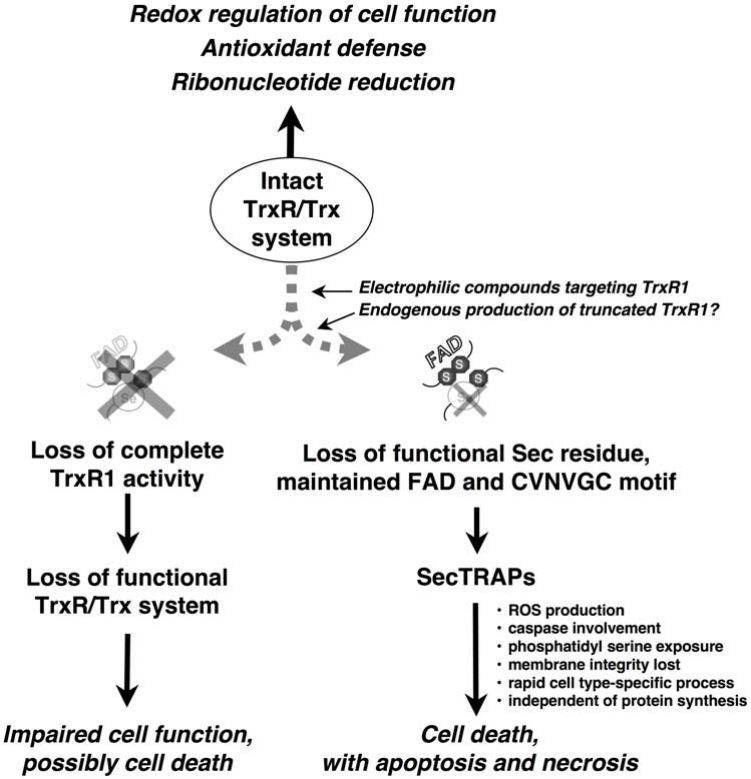
Model for the formation and function of SecTRAPs. We propose that during most conditions of normal cell growth the Sec residue in TrxR1 is intact and the role of this enzyme is thus to sustain the many cellular functions of the thioredoxin system. This activity is dependent upon the redox active C-terminal Sec-containing active site in TrxR1. SecTRAPs may however be formed from TrxR1 if its Sec residue becomes compromised, either by removal or by derivatization with electrophilic compounds, while the FAD and N-terminal redox active CVNVGC motif of the enzyme are kept intact. SecTRAPs lack the Trx reducing activity of native TrxR1 but become potent inducers of cell death by a gain of function. This cell death is, as shown in the present study, rapid, does not require induction of protein synthesis, involves production of reactive oxygen species and is prevented by caspase inhibitors. If, on the other hand, both the CVNVGC motif and the C-terminal selenolthiol motif become inactivated, e.g. by certain types of TrxR1 inhibitors or if the overall expression of TrxR1 is diminished in cells, impaired cell function or cell death may still occur. In such cases the cellular consequences would however not be due to a gain of function in derivatives of TrxR1, but rather to hampered functions of the complete cellular thioredoxin system.

Here we showed that SecTRAPs, devoid of the natural Sec-containing active site of TrxR1, could still have their FAD cofactor efficiently reduced by NADPH. Upon reduction by NADPH, SecTRAPs could also form the charge-transfer complex between the FAD moiety and the CVNVGC redox active disulfide/dithiol-containing site. The fact that the cell-killing SecTRAP properties evidently depend upon an intact FAD/CVNVGC-containing redox active motif and correlate to a prooxidant capacity has additional implications. A potential drug or compound that would inhibit the redox activity of this N-terminal motif, and thereby block the reductive half reaction of TrxR1-derived protein, would according to his notion *not* give rise to the formation of SecTRAPs. However, such potential drugs may still have major effects on cellular function as a result of the lowered capacity of the thioredoxin system, reminiscent to the effects of knocking down TrxR1 expression by siRNA [60], [68], [74], [75]. This emphasizes the delicate balance between different cellular effects that can be governed by either an intact thioredoxin system, by a loss of thioredoxin reductase activity, or by the formation of SecTRAPs. This concept is summarized in the scheme shown in Figure 9.

The pronounced protection of the cells by inhibitors of caspase-3/7 or caspase-2, and partially caspase-8, indicated that the apoptotic machinery was required for cell death to occur in connection with the oxidative stress triggered by SecTRAPs. The general features of the cell death found here, with both caspase-3 and caspase-8 involvement and signs of both apoptosis and necrosis, are in fact typical for cell death induced by oxidative stress [76]. Regarding caspase-2, recent findings have shown that this caspase when activated can induce apoptosis by directly promoting the release of cytochrome *c* from mitochondria [77], [78]. We have attempted to detect mitochondrial release of cytochrome *c* after treatment with SecTRAPs, however with inconclusive results. It should also be noted that it was recently shown that Ac-VDVAD-CHO, commonly used as caspase-2 inhibitor, may also inhibit caspase-3 [79]. Moreover, we also studied the effects of a caspase-9 inhibitor (z-LEHD-fmk) but with intermediate and inconclusive results (not shown). Therefore, taken together we do not know at present to which extent the SecTRAP-induced cell death involves caspase-2 activation, release of cytochrome *c* from the mitochondria or caspase-9 activation. We have been limited in our analyses by the low amount of cells used in experiments employing the *BioPORTER* protein delivery approach. We have attempted to construct stably transfected HeLa cells for conditional overexpression of the two-amino acid truncated TrxR1 but were not successful to do so, potentially because of the toxic effects of a low basal expression of truncated TrxR1 (S. Prast-Nielsen and E. Arnér, unpublished results). Future studies with other approaches are clearly needed for more detailed analyses of signaling events such as cytochrome *c* release from the mitochondria or direct detection of caspase activation.

Based upon the results presented in this study, we conclude that SecTRAPs, produced by C-terminal truncation of TrxR1 or by chemical derivatization of its Sec residue, are potent inducers of a cell death that involves both apoptotic and necrotic features in line with increased intracellular ROS production. The effect was rapid and did not require induction of protein synthesis. The properties of SecTRAPs obviously add another level of complexity to the thioredoxin system. It is becoming increasingly evident that the thioredoxin system is of major importance for cancer development and as a target for anticancer therapy [6], [64], [66]. Within that context, it is plausible that formation of SecTRAPs may play a role for some of the observed effects e.g. seen upon use of alkylating drugs in cancer treatment. Further studies are needed in order to fully understand both the exact molecular mechanisms by which SecTRAPs trigger apoptosis in cells and the potential physiological or clinical importance of the formation of these proteins.
